# “Short” is not always “scientific”: cross-sectional quality assessment and machine learning–based evaluation of weight management short videos on TikTok and Bilibili

**DOI:** 10.3389/fpubh.2026.1830047

**Published:** 2026-06-02

**Authors:** Jinsong Du, Le Zhu, Hailing Zhou

**Affiliations:** School of Health Management, Zaozhuang University, Zaozhuang, China

**Keywords:** health communication, information quality, machine learning, short video, weight management

## Abstract

**Objective:**

This study aimed to systematically evaluate and compare the content quality and reliability of weight management–related short videos on TikTok and Bilibili, identify key factors influencing video quality, and develop an automated quality prediction tool to support the construction of a healthier digital health communication ecosystem.

**Methods:**

A cross-sectional study design was adopted. The top 100 weight management–related videos ranked by overall relevance were collected from TikTok and Bilibili, respectively (total *n =* 200). Two independent researchers evaluated video quality and information reliability using the Global Quality Score (GQS) and the DISCERN instrument. Spearman’s rank correlation and Poisson regression analyses were conducted to explore associations between video characteristics and evaluation scores. An XGBoost-based model was developed to predict high-quality videos, and the SHAP framework was applied to interpret the model’s decision-making mechanism.

**Results:**

Videos on Bilibili had significantly higher GQS scores than those on TikTok (*p <* 0.001). However, DISCERN scores were generally low on both platforms, with no statistically significant difference. On TikTok, most videos were uploaded by Nonprofessional individuals and primarily focused on Personal experience sharing (68%), whereas the distribution of uploader sources on Bilibili was relatively balanced. Videos published by Professional individuals demonstrated significantly higher quality and reliability than those published by Nonprofessional individuals. Regression analyses indicated that video duration and number of likes were positive predictors of both quality and reliability. The XGBoost model achieved good discriminative performance in the test set (AUC = 0.8072). SHAP analysis revealed that when video duration exceeded 12.75 min, its contribution to predicting high-quality videos shifted from negative to positive. Based on these findings, an accessible online evaluation platform for high-quality short videos was developed and deployed.

**Conclusion:**

Videos published by Professional individuals possess greater academic value and practical significance. However, weight management information on short-video platforms exhibits a mismatch between popularity and quality. The automated evaluation model and online tool proposed in this study provide strong support for the public in identifying reliable scientific information and for regulatory authorities in developing intelligent governance systems.

## 1 Introduction

Obesity has become one of the most challenging global public health issues of the 21st century and is closely associated with an increased risk of cardiovascular diseases, diabetes, and various chronic non-communicable diseases (1–3). Active and evidence-based weight management is a key strategy for preventing and mitigating obesity and its related complications (4–6). With the widespread adoption of mobile internet technologies, the way the public accesses health information has undergone a fundamental transformation (7, 8). In China, short-video platforms represented by TikTok and Bilibili have rapidly emerged as primary channels for obtaining weight management advice due to their strong interactivity, intuitive visual presentation, and algorithm-driven personalized recommendations. These platforms demonstrate considerable potential in breaking down barriers to professional knowledge and lowering the threshold for medical knowledge popularization, attracting a vast number of users to participate in in-depth discussions on fitness practices and dietary interventions.

However, the openness of short-video platforms has also introduced substantial concerns regarding content quality. In the absence of rigorous medical review mechanisms at the production level, platforms are inundated with fragmented information published by Nonprofessional individuals (9–11). Such content often emphasizes personal testimonials or emotionally driven Personal experience sharing rather than arguments grounded in scientific evidence. Previous studies have observed a frequent disconnect between engagement metrics—such as likes and favorites—and the medical reliability of videos on social media platforms (12–14). This traffic driven logic prioritized content ecosystem over medical logic not only increases the difficulty for the public to identify scientifically valid information, but may also mislead users into adopting unscientific, irregular, or even harmful weight-loss behaviors, potentially resulting in irreversible health consequences. Although research on health communication through social media is expanding, identifying truly high-quality content with knowledge popularization value from massive volumes of interactive data and uncovering the core determinants driving video quality remain critical challenges for improving digital health communication. Traditional regulatory approaches struggle to cope with the vast and dynamic flow of information, underscoring the urgent need for systematic and scientific evaluation frameworks to deconstruct the landscape of health communication in the short-video era.

Using a cross-sectional study design, this study aimed to systematically evaluate and compare the quality characteristics and reliability of weight management–related short videos on TikTok and Bilibili. Standardized assessments were conducted using the Global Quality Score (GQS) and the DISCERN instrument. In addition, machine learning techniques, specifically XGBoost, were employed alongside the SHAP framework to identify key factors driving video quality. The significance of this study lies in revealing inter-platform differences in weight management–related short videos and in developing an accessible online predictive platform to provide scientific evaluation tools for both the public and regulatory authorities, thereby contributing to the establishment of a more transparent and credible short-video health information ecosystem.

## 2 Methods

### 2.1 Ethics review

This study strictly adheres to academic ethical standards and the principles of anonymity and privacy protection as outlined in the Declaration of Helsinki. All research data were sourced from public videos on social media platforms such as TikTok and Bilibili, and the data collection process strictly complied with the terms of service of each platform. This study exclusively analyzed publicly available audio-visual content and interaction data; throughout the research, all data underwent de-identification and anonymization, involving no clinical patient data, human tissue samples, or any private personal information. Additionally, this study is categorized as a passive observational study, with no interactions or experimental interventions conducted with video creators or platform users. The research was reviewed and approved by the Research Ethics Committee of Zaozhuang University (ethics review number: UZZ-PP-2026001).

### 2.2 Data collection

This study adopted a cross-sectional design to systematically evaluate the quality, reliability, and predictive models of weight management–related short videos on two major short-video platforms in China, TikTok and Bilibili. On February 10, 2026, the keyword “weight management” was used to systematically search for relevant videos on both platforms for content analysis.

To minimize potential search bias caused by personalized recommendation algorithms, newly registered accounts were created and used to log into each platform before conducting the formal search. Based on the platforms’ comprehensive ranking results, the top 100 videos from each platform were selected as the study sample. Multiple studies have confirmed that videos ranked beyond the top 100 have no significant impact on analytical outcomes (15, 16). The platform ranking algorithms typically integrate multiple engagement metrics, including watch completion rate, likes, comments, and shares. Therefore, the selected sample can, to some extent, represent content that users are frequently exposed to and that has a relatively wide dissemination range.

**Figure 1:**
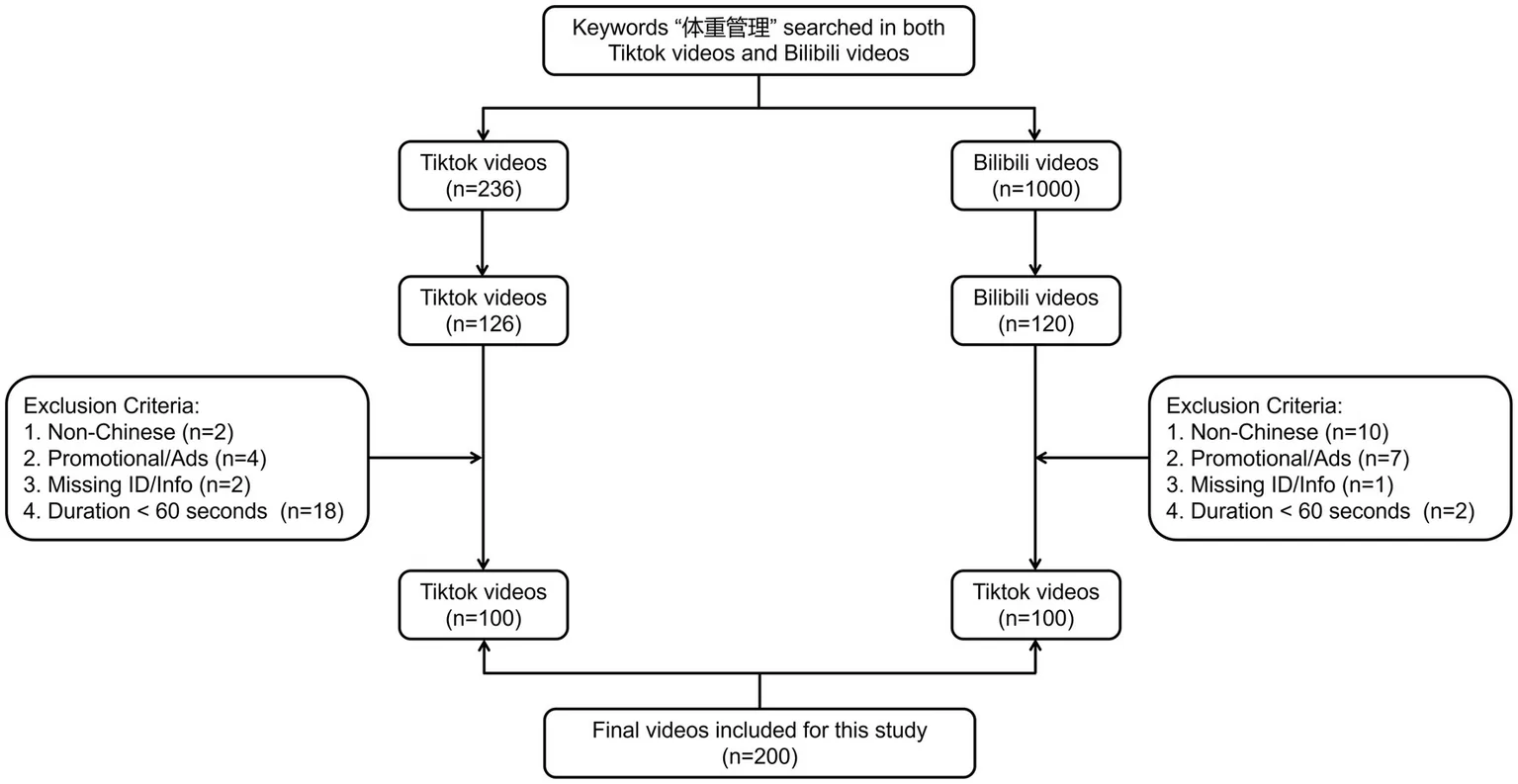
Search strategy for weight management short videos.

During the screening process, videos were strictly excluded according to the following criteria (Figure 1): (1) Non-Chinese-language videos; (2) Advertisements or marketing-oriented videos unrelated to the research topic; (3) Videos lacking clear creator identity information or with incomplete titles; (4) Videos with a duration of less than 60 s, as such videos are generally limited by time constraints and are unlikely to provide substantial medical or health information. The screening process continued until the top 100 eligible videos from each platform were obtained. This inclusion range was based on commonly used methods in previous related studies, which indicate that videos ranked beyond 100 have limited influence on overall analytical results. For each included video, the following basic information was recorded: platform source, video title, uploader name and identity, video content, video duration, number of likes, number of comments, number of shares, and number of favorites.

### 2.3 Video classification

Videos were categorized into four groups according to their source: (1) Professional individuals; (2) Nonprofessional individuals; (3) Professional institutions; (4) Nonprofessional institutions. Among videos published by Professional individuals, further classification was conducted into Medical Specialists and Other Healthcare Professionals, such as nutritionists and fitness coaches. According to video content, the videos were classified into the following categories: (1) Knowledge popularization; (2) Practical guidance on diet and exercise; (3) Personal experience sharing on weight management; (4) Related policies and news information.

### 2.4 Assessment of video quality and information reliability

The Global Quality Score (GQS) was used to evaluate the overall informational quality of the videos, and the DISCERN instrument was used to assess the reliability of the health information provided. The GQS scoring system consists of five levels, with scores ranging from 1 to 5; higher scores indicate higher video quality (17). The DISCERN tool is used to evaluate the credibility of health information, with higher scores indicating more reliable information sources (18). The detailed scoring criteria and grading methods of GQS and DISCERN are presented in Supplementary Tables S1–S3.

All included videos were uniformly collected and saved by one researcher, after which the order of the videos was randomized and independently evaluated by two health management professionals. Before the formal evaluation, both evaluators jointly studied and discussed the scoring rules of GQS and DISCERN to unify their understanding of the criteria and reduce the influence of subjective differences. When discrepancies occurred between the two evaluators for the same video, a third research member reviewed the case and determined the final score. Ultimately, consensus on all scores was reached among the research team members. In addition, Cohen’s *κ* coefficient was used to assess inter-rater agreement between the two evaluators. A κ value greater than 0.80 indicates excellent agreement; 0.60–0.80 indicates good agreement; 0.40–0.60 indicates moderate agreement; and below 0.40 indicates poor agreement (19).

### 2.5 Machine learning model

In this study, a predictive model based on the XGBoost algorithm was constructed to evaluate short-video quality. Nine features, including video duration and number of favorites, were used as input variables to predict whether a video belonged to the high-quality category. High-quality videos were defined as those with both GQS and mDISCERN scores ≥ 4. The samples were randomly divided into a training set and a test set at a ratio of 7:3, and hyperparameters were optimized using grid search combined with 10-fold cross-validation. The classification threshold of the model was determined based on the receiver operating characteristic curve, and the optimal cutoff point was selected by maximizing the Youden index to achieve the best balance between sensitivity and specificity. The SHAP framework was introduced to interpret the model’s prediction logic by calculating feature contribution values to quantify the impact of each indicator on quality prediction (20–22). In addition, an online evaluation system was established using a Tencent Cloud server^1^ to visualize the research results and enable real-time prediction.

### 2.6 Statistical analysis

All statistical analyses were performed in Python 3.11. As the data were non-normally distributed, continuous variables were described using the median (interquartile range). Differences between two groups were compared using the Mann–Whitney U test, while differences among multiple groups were analyzed using the Kruskal–Wallis test, followed by Dunn’s multiple comparison test for pairwise comparisons. Inter-rater agreement between the two evaluators was assessed using Cohen’s *κ* coefficient, and correlations between variables were analyzed using Spearman’s rank correlation. In addition, although GQS and DISCERN scores are inherently ordinal variables, they can be approximately treated as count-type outcomes due to their relatively limited range. Therefore, a Poisson regression model was employed to assess the association between video-related characteristics and quality scores. All statistical tests were two-sided, and *p <* 0.05 was considered statistically significant.

## 3 Results

### 3.1 Video characteristics

A total of 200 weight management–related short videos were included in this study, comprising 100 videos from TikTok (*n =* 100) and 100 videos from Bilibili (*n =* 100). A comparison of video characteristics between the two platforms is presented in Table 1. In terms of engagement performance, TikTok videos had significantly higher numbers of likes, comments, and shares than Bilibili videos (all *p <* 0.05). Although the number of favorites on TikTok was also higher than that on Bilibili, the difference between the two groups did not reach statistical significance (*p* = 0.89). In contrast, videos on Bilibili had significantly longer durations and longer publication days compared with those on TikTok (*p <* 0.001).

**Table 1:** Comparison of video characteristics between TikTok and Bilibili platforms.

Categories	TikTok (*n =* 100)	Bilibili (*n =* 100)	Mann–Whitney U	
			*z-*value	*p*-value
Likes	24,000 (3108.25–147,250)	8673.5 (1046.50–34,000)	−3.07	<0.01
Comments	816 (145.25–6740.5)	472 (95.75–2484.75)	−2.37	<0.05
Shares	4,791 (515.25–31,000)	1,147 (214.25–9,827)	−2.76	<0.01
Saves	8,669 (1120.5–54,500)	7,610 (1276–35,250)	−0.14	0.89
Duration	2.4 (1.73–4)	10.17 (4.27–16.36)	8.42	<0.001
Days	108.5 (64.5–175)	684.50 (199.25–1484.25)	7.60	<0.001
GQS	2 (2–3)	3 (2–4)	3.53	<0.001
DISCERN	3 (2–4)	3 (2–4)	0.35	0.726

**Figure 2:**
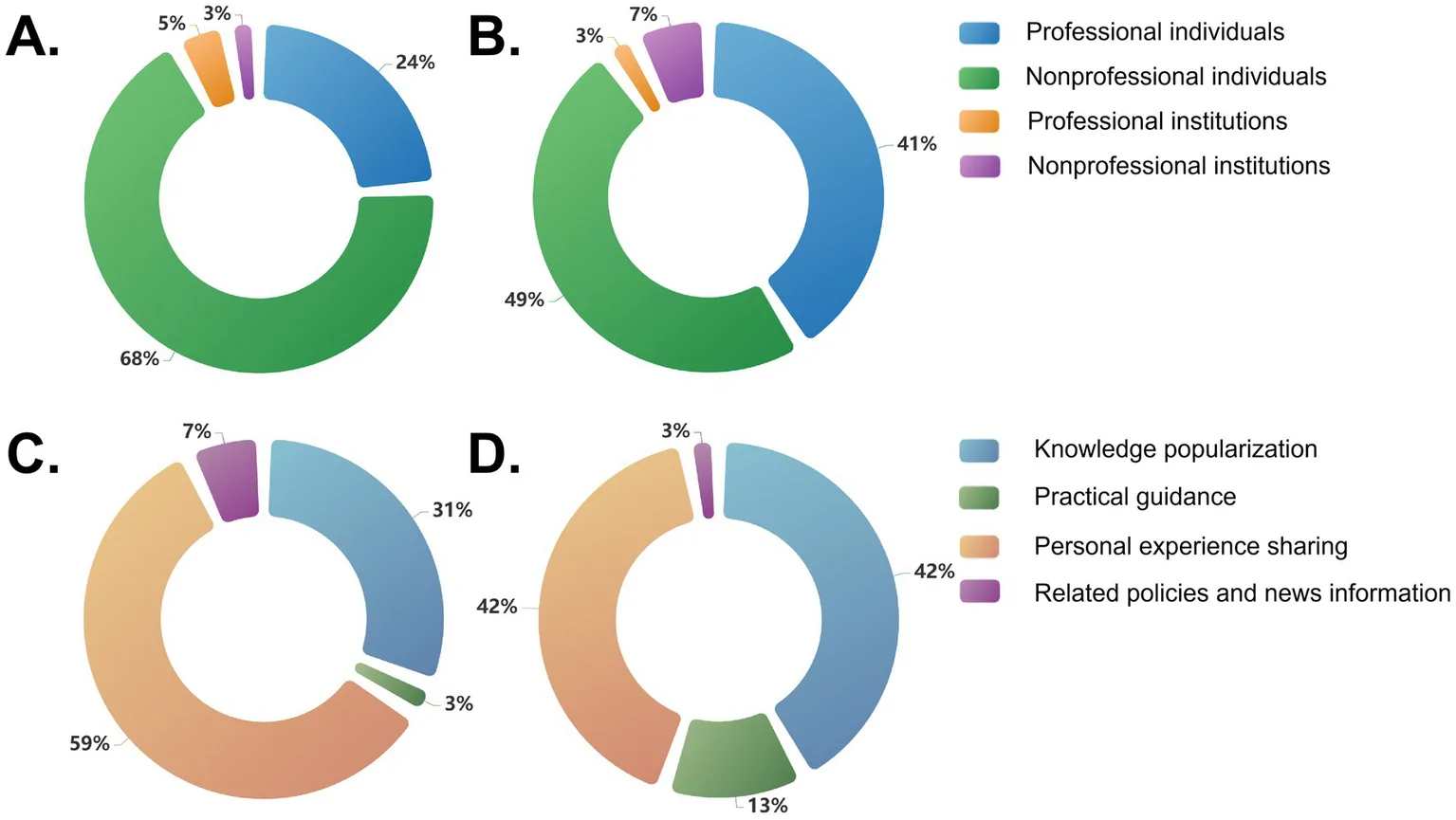
Distribution of uploader sources and content categories of weight management–related short videos on TikTok and Bilibili. **(A)** Distribution of video uploader sources on TikTok; **(B)** distribution of video uploader sources on Bilibili; **(C)** classification of video content categories on TikTok; **(D)** classification of video content categories on Bilibili.

As shown in Figure 2 and Supplementary Table S4, TikTok and Bilibili exhibited distinct patterns in terms of uploader sources and content categories. On TikTok, Nonprofessional individuals were the primary contributors, accounting for 68% (*n =* 68) of all videos. This was followed by Professional individuals in the medical field, representing 24% (*n =* 24). Professional institutions and Nonprofessional institutions accounted for 5% (*n =* 5) and 3% (*n =* 3), respectively. In terms of content categories, TikTok videos were predominantly focused on Personal experience sharing (*n =* 59, 59%), followed by Knowledge popularization (*n =* 31, 31%), Related policies and news information (*n =* 7, 7%), and Practical guidance (*n =* 3, 3%). In contrast, the distribution of uploader sources on Bilibili was relatively balanced. Although Nonprofessional individuals still constituted the largest proportion (*n =* 49, 49%), Professional individuals in the medical field accounted for a substantial share of 41% (*n =* 41). Regarding content categories, Bilibili placed equal emphasis on Knowledge popularization and Personal experience sharing, each accounting for 42% (*n =* 42).

### 3.2 Video quality and reliability assessment

**Figure 3:**
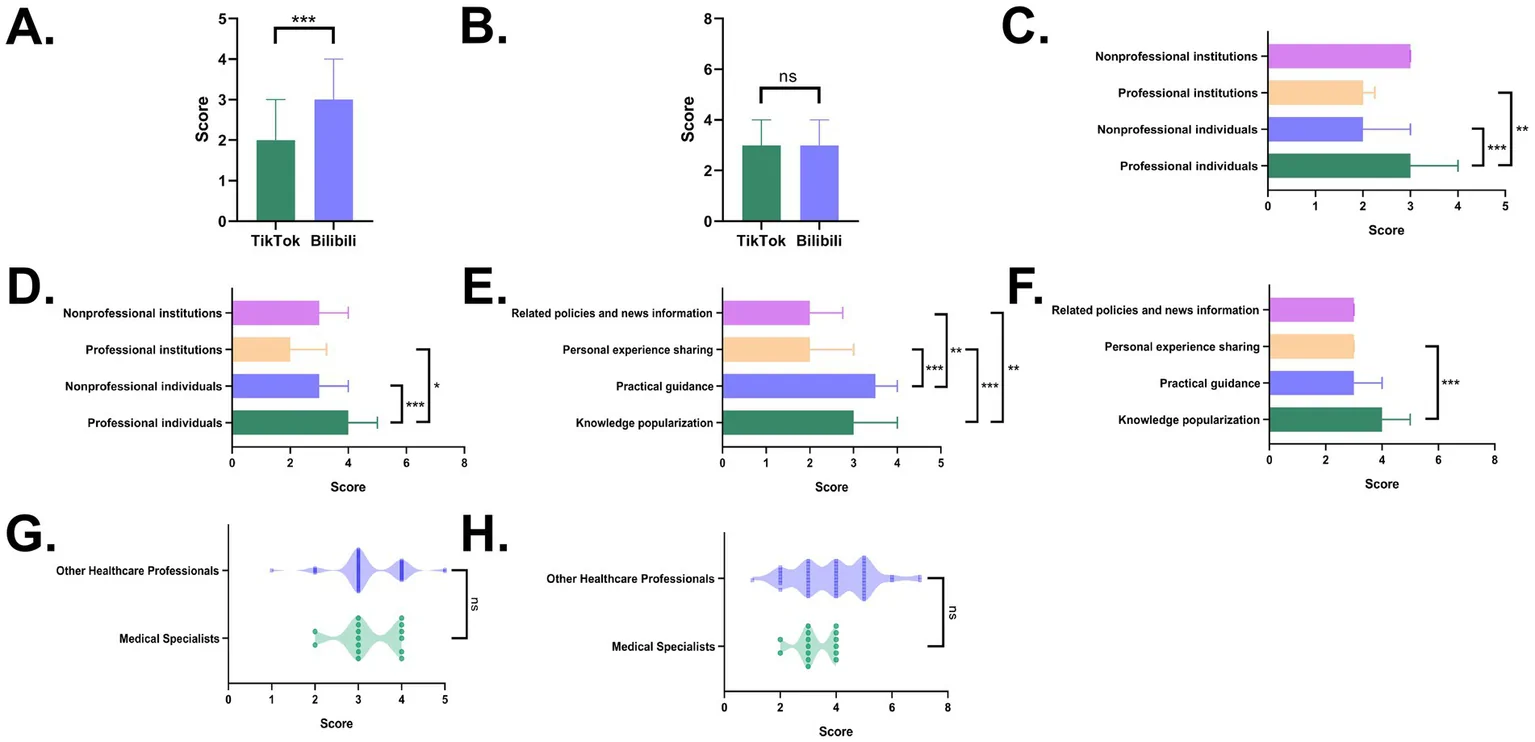
Score analysis of weight management–related short videos on TikTok and Bilibili. **(A)** Comparison of GQS scores between TikTok and Bilibili videos; **(B)** comparison of DISCERN scores between TikTok and Bilibili videos; **(C)** distribution of GQS scores across different uploader sources; **(D)** distribution of DISCERN scores across different uploader sources; **(E)** distribution of GQS scores across different content categories; **(F)** distribution of DISCERN scores across different content categories; **(G)** GQS scores of videos uploaded by different medical professional backgrounds; **(H)** DISCERN scores of videos uploaded by different medical professional backgrounds.

The inter-observer agreement was good, with a *κ* value of 0.77 (95% CI: 0.71–0.83). Figures 3A,B and Supplementary Table S5 present the distribution of GQS and DISCERN scores for videos on TikTok and Bilibili. On TikTok, the median GQS score was 2 (IQR: 2–3), and the median DISCERN score was 3 (IQR: 2–4), indicating that the overall quality and reliability of videos on this platform were relatively low. In contrast, videos on Bilibili had a median GQS score of 3 (IQR: 2–4) and a median DISCERN score of 3 (IQR: 2–4), suggesting moderate quality but low reliability. Statistical analysis showed a significant difference in GQS scores between TikTok and Bilibili (*p <* 0.001), whereas no statistically significant difference was observed in DISCERN scores (*p* = 0.726). These findings indicate that although Bilibili videos demonstrated higher quality than TikTok videos, the reliability of videos on both platforms requires improvement.

This study further compared GQS and DISCERN scores across different uploader sources and content categories on the two platforms (Figures 3C–F). Regarding uploader sources, videos produced by Professional individuals had significantly higher GQS scores than those produced by Nonprofessional individuals (*p <* 0.001) and Professional institutions (*p <* 0.01). In terms of reliability, videos produced by Professional individuals also had significantly higher DISCERN scores than those produced by Nonprofessional individuals (*p <* 0.001) and Professional institutions (*p <* 0.05). These results suggest that medical professionals provide weight management information with higher quality and reliability. Regarding content categories, significant differences in quality were observed among different types. Knowledge popularization videos had significantly higher GQS scores than Personal experience sharing (*p <* 0.001) and related policies and news information (*p <* 0.01). Meanwhile, Practical guidance videos also had significantly higher GQS scores than Personal experience sharing (*p <* 0.001) and related policies and news information (*p <* 0.001). In terms of reliability, Knowledge popularization videos had significantly higher DISCERN scores than Personal experience sharing videos (*p <* 0.001). These findings suggest that videos focusing on theoretical knowledge popularization and practical guidance are generally more valuable and informative than those based on personal experiences or news dissemination.

To further explore whether different types of professionals influenced video quality and reliability, videos uploaded by Professional individuals were subdivided into Medical Specialists and Other Healthcare Professionals. The results showed no statistically significant differences between the two groups in terms of GQS scores (*p* = 0.814) or DISCERN scores (*p* = 0.879).

### 3.3 Correlation and Poisson regression analyses

**Figure 4:**
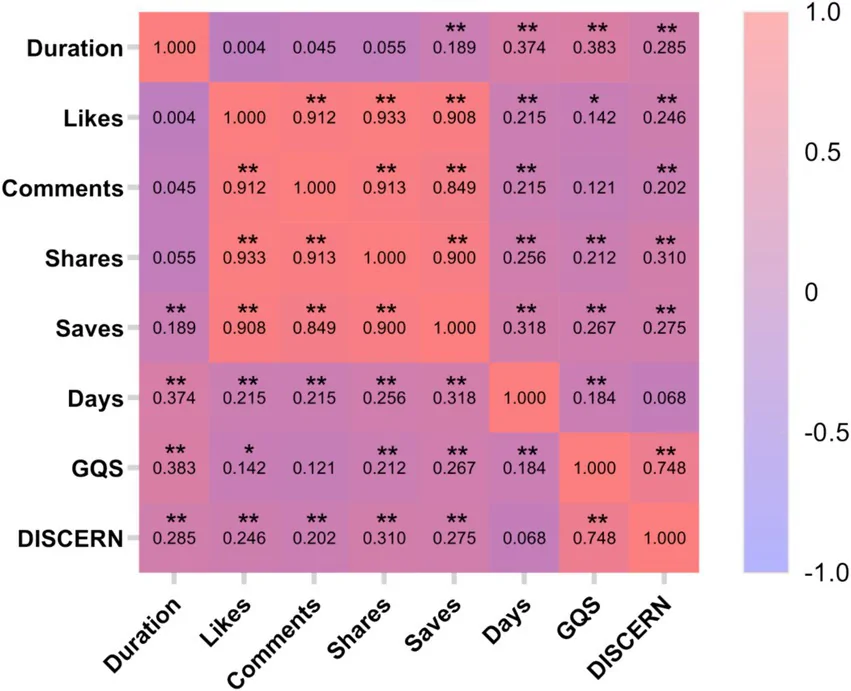
Correlation analysis heatmap.

Spearman’s rank correlation analysis was performed to examine the relationships between video characteristics and evaluation scores (Figure 4). The results showed that video duration was significantly positively correlated with both GQS scores (*p <* 0.01) and DISCERN scores (*p <* 0.01). Regarding engagement metrics, the number of likes, shares, and saves were significantly positively correlated with GQS scores (*p <* 0.05, *p <* 0.01, and *p <* 0.01, respectively) and DISCERN scores (all *p <* 0.01). Poisson regression analysis was conducted to assess the associations between video characteristics and evaluation scores (Table 2). The results indicated that both the number of likes and video duration were significant positive predictors of GQS and DISCERN scores (Table 2).

**Table 2:** Associations between video variables and GQS and DISCERN scores.

Variables	GQS	DISCERN
Likes Exp(B) (95% CI)	1.0000001 (1.000000–1.000002)***	1.000001 (1.000000–1.000002)*
Saves Exp(B) (95% CI)	1.000000 (1.000000–1.000001)	1.000000 (1.000000–1.000001)
Comments Exp(B) (95% CI)	0.999995 (0.999988–1.000002)	0.999995 (0.999986–1.000004)
Shares Exp(B) (95% CI)	0.999999 (0.999997–1.000001)	1.000000 (0.999998–1.000003)
Duration Exp(B) (95% CI)	1.000005 (1.000002–1.000008)**	1.000007 (1.000003–1.000011)**
Days Exp(B) (95% CI)	1.000000 (1.000000–1.000000)	1.000000 (1.000000–1.000000)

*Indicates *p* < 0.05, **p < 0.01, and ***p < 0.001.

### 3.4 Feature importance analysis

**Figure 5:**
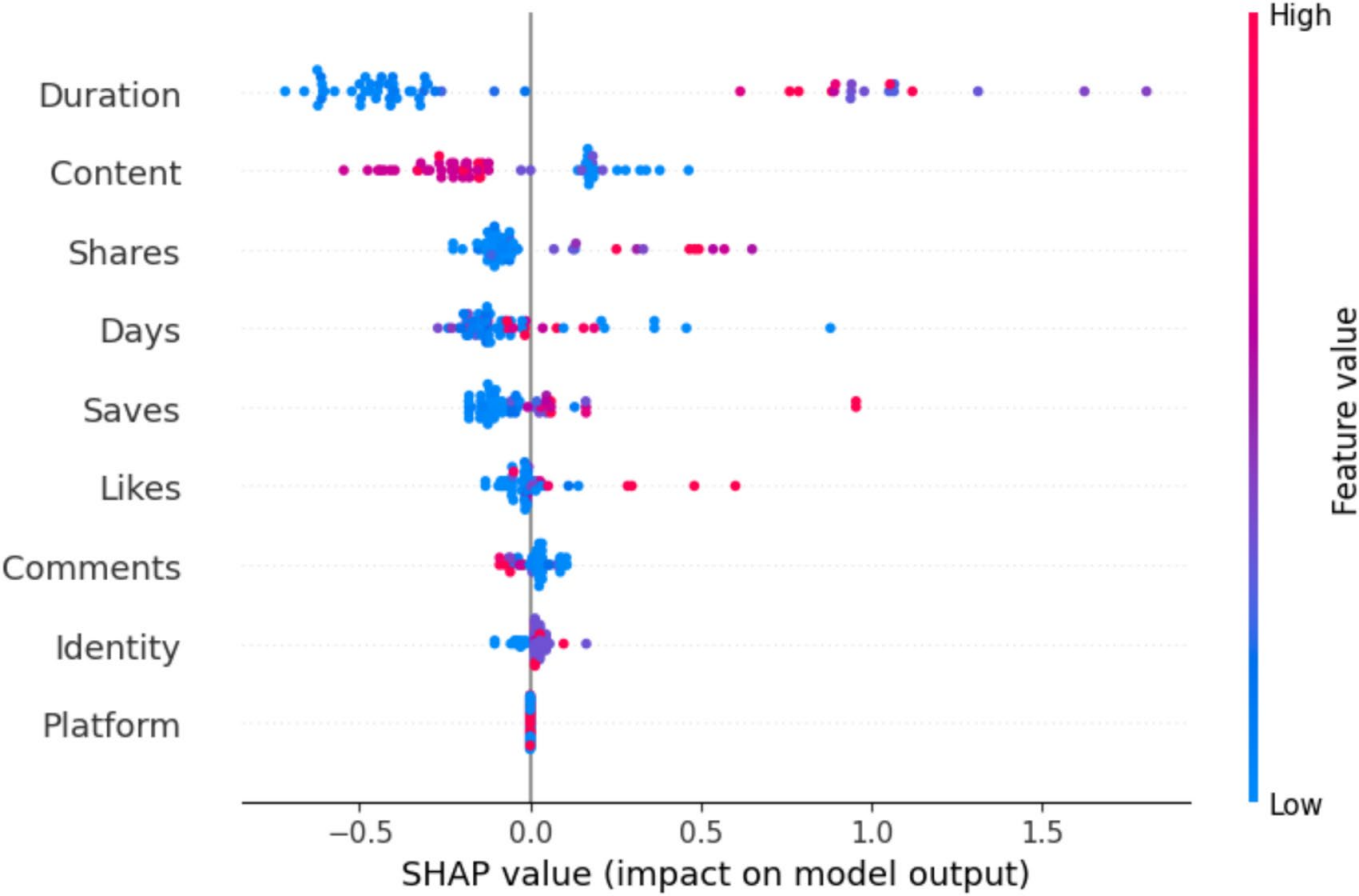
SHAP analysis plot.

Machine learning algorithms were further applied to explore the association between video characteristics and high-quality weight management videos. High-quality videos were defined as those with both GQS and DISCERN scores ≥ 4, resulting in 29 eligible samples. The XGBoost-based predictive model demonstrated good discriminative performance in the test set, with an area under the curve (AUC) of 0.8072 (Supplementary Figure S1). The SHAP framework was used to interpret the model’s decision-making logic (Figure 5). The summary plot indicated that video duration was the most important feature contributing to the prediction of high-quality videos. In addition, content category, number of shares, days since publication, and number of saves also showed relatively high feature importance, jointly driving the model’s identification of high-quality content. Further analysis using SHAP dependence plots (Supplementary Figure S2) revealed a nonlinear trend in the key variable. Video duration exhibited a clear threshold effect on prediction results. When video duration reached 12.75 min, its SHAP contribution value shifted from negative to positive.

### 3.5 Construction of the online prediction model

**Figure 6:**
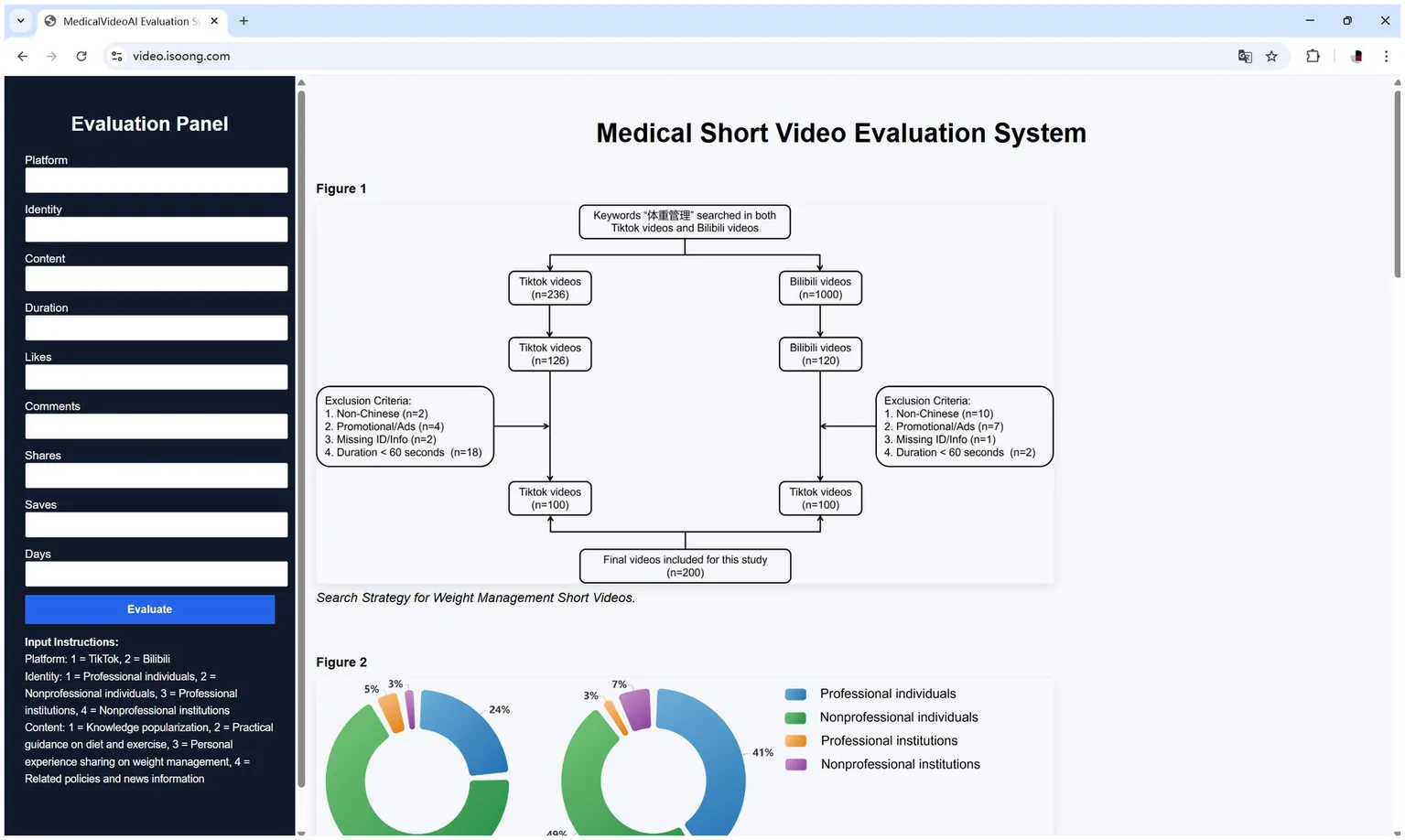
Online evaluation platform (https://video.isoong.com).

To promote the translation and open access of the research findings, an interactive online prediction platform was developed and deployed based on Tencent Cloud architecture (see text footnote 1). As shown in Figure 6, the platform integrates the above-mentioned XGBoost predictive model, allowing users and content creators to input video parameters for real-time quality evaluation, while simultaneously displaying the analytical results of this study, providing intuitive data support for understanding the characteristics of high-quality medical knowledge popularization content.

## 4 Discussion

Short-video platforms have become one of the primary channels for the public to obtain weight management information; however, the scientific rigor and reliability of their content still face significant challenges. This study adopted a cross-sectional design to systematically evaluate content from two major short-video platforms, TikTok and Bilibili. It should be noted that the term “short video” in this study is defined based on platform characteristics and content dissemination format, rather than a strict duration threshold. To ensure that included videos had sufficient informational content, videos shorter than 60 s were excluded. The results showed that Bilibili videos had significantly higher GQS scores than TikTok videos. This discrepancy may be attributed to differences in platform positioning and user ecosystems. TikTok is characterized by a “fast-paced, high-entertainment” model (23, 24), with generally shorter video durations (median: 2.4 min), which often leads to fragmented information delivery and limits the in-depth presentation of complex medical logic. In contrast, videos on Bilibili had substantially longer durations (median: 10.17 min), providing greater structural space for systematic and comprehensive knowledge popularization. Nevertheless, DISCERN scores on both platforms were relatively low (median *=* 3 for both), with no statistically significant difference observed. This suggests that although some videos may perform moderately well in narrative presentation, both platforms exhibit common deficiencies in key reliability dimensions, such as evidence citation and comprehensive evaluation of treatment options. These findings indicate that current weight management short videos remain in a stage characterized by “emphasis on presentation over depth,” highlighting the urgent need for more professional content entry and dissemination mechanisms.

The results further confirm that uploader professional background and content category are important determinants of video quality. Videos produced by Professional individuals achieved significantly higher GQS and DISCERN scores than those produced by Nonprofessional individuals and institutions, reinforcing the foundational role of professional medical expertise in health communication (18, 25, 26). Interestingly, within the subgroup of professionals, no significant difference was observed between physicians and other healthcare providers, suggesting that in the interdisciplinary field of weight management, individuals with systematic professional knowledge can produce similarly high-quality content. However, on TikTok, as many as 68% of videos were uploaded by Nonprofessional individuals, and most content consisted of fragmented “Personal experience sharing.” This tendency toward personal testimony rather than scientific argumentation may increase the risk of misinformation and potentially lead the public to overlook evidence-based principles of weight management. In contrast, Knowledge popularization and Practical guidance videos demonstrated greater reference value and may more effectively enhance public health literacy.

Spearman correlation analysis and Poisson regression analysis both indicated that video duration was a positive predictor of quality scores. Although the number of likes was also positively correlated with evaluation scores, its regression coefficient was relatively small. This may reflect the tendency for highly engaging or emotionally stimulating nonprofessional videos to accumulate higher interaction metrics. Such a mismatch between quality and popularity creates a scenario in which users may mistakenly infer credibility based on engagement indicators such as likes and comments. Therefore, the public should remain cautious when evaluating highly liked weight management videos and avoid using popularity as the sole indicator of quality.

Machine learning models provide new technical possibilities for automated short-video quality governance. The XGBoost model constructed in this study achieved an AUC of 0.8072 in the test set, demonstrating strong discriminative performance. The SHAP framework further quantified the contribution of each feature to model predictions, identifying video duration as the dominant factor in predicting high-quality content. Notably, the SHAP dependence plot revealed a threshold effect of video duration on content quality. In this dataset, when the duration exceeded 12.75 min, its contribution to predicting high-quality videos shifted from negative to positive. Based on these findings, platform operators could use such algorithms to automatically prioritize high-quality Knowledge popularization content or issue early warnings for potentially high-risk content. Furthermore, by establishing an online evaluation system, this study enhances practical applicability and fills the gap in quality self-assessment tools currently lacking on short-video platforms.

However, several limitations should be acknowledged. First, the cross-sectional design only captures data at a specific time point and cannot fully reflect the dynamic evolution of short-video trends. Moreover, the prediction model constructed based on a single time point without external validation may have limited stability and generalizability. Second, although inter-rater reliability was ensured through multiple reviewers and Cohen’s *κ* coefficient, some subjectivity in GQS and DISCERN scoring cannot be completely eliminated. Finally, due to the relatively small number of high-quality videos, class imbalance may have been introduced, potentially affecting model stability. Therefore, although the XGBoost model demonstrated certain discriminatory ability in the current dataset, its performance under different data distributions still requires further validation.

## 5 Conclusion

In summary, this study systematically evaluated the quality characteristics of weight management–related videos on TikTok and Bilibili and developed an effective automated assessment model. The results indicate that, within this dataset, professionally produced longer videos exceeding 12.75 min were associated with higher quality and reliability. This study not only highlights the discrepancy between engagement metrics and medical depth but also provides a practical evaluation tool for the public and regulatory authorities through an online platform developed based on Tencent Cloud architecture. Improving the short-video health communication environment requires the combined efforts of professional content support, scientifically optimized platform algorithms, and enhanced media literacy among the public.

## Data Availability

The raw data supporting the conclusions of this article will be made available by the authors, without undue reservation.

## Footnotes

1
https://video.isoong.com
