# Correction: “Short” is not always “scientific”: cross-sectional quality assessment and machine learning–based evaluation of weight management short videos on TikTok and Bilibili

**DOI:** 10.3389/fpubh.2026.1980796

**Published:** 2026-09-08

**Authors:** Frontiers Production Office

**Affiliations:** Frontiers Media SA, Lausanne, Switzerland

**Keywords:** health communication, information quality, machine learning, short video, weight management

Author “Hailing Zhou” was erroneously assigned as equal contributing author.

The original version of this article has been updated.

