# Supplementary material for: “Short” is not always “scientific”: cross-sectional quality assessment and machine learning–based evaluation of weight management short videos on TikTok and Bilibili
Source: Front Public Health. 2026 Jun 2;14:1830047. doi: 10.3389/fpubh.2026.1830047 (PMC13292766; doi:10.3389/fpubh.2026.1830047)
Supplement: Supplementary file 1 [file Table_1.DOCX]

**Supplementary Materials**

Table S1. Global Quality Score (GQS) Criteria for Evaluating the Quality of Weight Management–Related Short Videos..

| Score | Description |
| --- | --- |
| 1 | Very poor video quality with disorganized logic; critical information is severely lacking and the video has no reference value for the audience. |
| 2 | Overall low quality with poor content coherence; contains limited information but lacks key points, with extremely low practical value. |
| 3 | Moderate quality with average structure; some core content is adequately addressed, but overall coverage is incomplete and has limited reference value. |
| 4 | Good quality with clear logic; covers most key information, with only minor omissions, and is beneficial to the audience. |
| 5 | Excellent quality with fluent presentation; information is complete and accurate, providing strong decision-making reference value for the audience. |

Table S2. DISCERN Criteria for Evaluating the Reliability of Weight Management–Related Short Videos.

| Item | Description |
| --- | --- |
| Q1 | Is the purpose of the video clearly defined? |
| Q2 | Does the video achieve its stated objectives? |
| Q3 | Is the content targeted and relevant to the intended audience? |
| Q4 | Are information sources other than the author clearly stated? |
| Q5 | Are the publication dates or timeliness of the cited information clearly indicated? |
| Q6 | Is the content balanced, objective, and free from bias? |
| Q7 | Are additional support resources or further information pathways provided? |
| Q8 | Are existing controversies or uncertainties in the current medical field mentioned? |

Table S3. Five-Level Scoring System for GQS and DISCERN.

| Score | Grade |
| --- | --- |
| GQS |  |
| 1 | Very poor |
| 2 | Poor |
| 3 | Moderate |
| 4 | Good |
| 5 | Excellent |
| DISCERN |  |
| 1 | Very low reliability |
| 2-3 | Low reliability |
| 4-5 | Moderate reliability |
| 6-7 | High reliability |
| 8 | Very high reliability |

Table S4. Distribution of Video Characteristics Across Different Sources and Content Categories on TikTok and Bilibili.

|  | Likes | Comments | Shares | Saves | Duration | Days |
| --- | --- | --- | --- | --- | --- | --- |
| TikTok Video Sources (n = 100), Median (IQR) | | | | | | |
| Professional individuals  (n = 24) | 25000  (6548.5-161500) | 1908.5  (268.75-6791.5) | 6518.5  (1226.5-48250) | 10318.5  (3199.25-55500) | 2.87  (2.21-6) | 126  (77-199.25) |
| Nonprofessional individuals  (n = 68) | 34000  (3906.5-172500) | 1212  (206.75-7351.5) | 5185.5  (515.25-30250) | 12000  (1388.5-60000) | 2.3  (1.7-3.73) | 106  (60.75-147.25) |
| Professional institutions  (n = 5) | 219  (34-508) | 37  (10-102) | 201  (23-785) | 43  (9-185) | 1.65  (1.3-1.67) | 153  (95-155) |
| Nonprofessional institutions  (n = 3) | 12000  (8351.5-50000) | 193  (174-11596.5) | 1441  (1294.5-64220.5) | 2208  (1678-11104) | 4  (4-4.37) | 320  (260.5-328) |
| TikTok Video Content Categories (n = 100), Median (IQR) | | | | | | |
| Knowledge popularization  (n = 31) | 26000  (7600.5-146000) | 1855  (234-4780) | 5521  (1411-42500) | 13000  (3883-49000) | 2.37  (1.94-5.39) | 119  (77.5-182.5) |
| Practical guidance  (n = 3) | 48000  (36000-145500) | 1077  (776.5-2585.5) | 8220  (6203-24110) | 35000  (18038.5-64500) | 2.27  (1.8-3.14) | 104  (79-108.5) |
| Personal experience sharing  (n = 59) | 32000  (2840.5-175000) | 829  (154-8270.5) | 5159  (343.5-29000) | 9578  (943.5-62000) | 2.40 (1.72-3.88) | 108  (60.5-155) |
| Related policies and news information  (n = 7) | 622  (363.5-8351.5) | 140  (69.5-174) | 805  (493-1294.5) | 219  (114-1678) | 2.58  (1.66-4.00) | 201  (154-265) |
| Bilibili Video Sources (n = 100), Median (IQR) | | | | | | |
| Professional individuals  (n = 41) | 8227  (1715-51000) | 590  (98-3235) | 2166  (409-15000) | 14000  (1885-63000) | 11.52  (4.7-18.65) | 924  (176-1567) |
| Nonprofessional individuals  (n = 49) | 12000  (1630-39000) | 550  (152-2315) | 902  (217-8367) | 8167  (1363-35000) | 9.3  (5.62-14.67) | 816  (237-1382) |
| Professional institutions  (n = 3) | 86  (48-189) | 4  (3-9) | 299  (186.5-329.5) | 219  (130-908) | 3.05  (2.64-12.35) | 334  (285-1030.5) |
| Nonprofessional institutions  (n = 7) | 835  (615.5-3289.5) | 107  (36.5-278.5) | 200  (152-2847) | 539  (416.5-2709.5) | 2.58  (1.51-8.2) | 206  (183-462.5) |
| Bilibili Video Content Categories (n = 100), Median (IQR) | | | | | | |
| Knowledge popularization  (n = 42) | 3019.5  (607.25-30500) | 425.5  (50.5-2164.75) | 792  (114.25-6197.5) | 3496.5  (449.75-17000) | 8.11  (3.79-13.42) | 505  (177.5-1440.75) |
| Practical guidance  (n = 13) | 28000  (8215-118000) | 5900  (389-9181) | 14000  (2166-67000) | 64000  (23000-306000) | 15.97  (10-31.02) | 1388  (237-1960) |
| Personal experience sharing  (n = 42) | 11500  (3674.25-38500) | 578.5  (143-1688.75) | 935.5  (284.25-7932.25) | 8072.5  (3429.25-35000) | 10.36  (4.87-16.04) | 803  (202.25-1328) |
| Related policies and news information  (n = 3) | 1856  (971-3289.5) | 271  (137.5-764.5) | 2315  (1337.5-2847) | 2604  (1411.5-2709.5) | 2.22  (1.71-2.4) | 338  (336-462.5) |

Table S5. Comparison of GQS and DISCERN Scores Between TikTok and Bilibili Videos.

|  | TikTok | Bilibili |
| --- | --- | --- |
| GQS | | |
| 1 | 7 | 8 |
| 2 | 46 | 23 |
| 3 | 39 | 41 |
| 4 | 6 | 25 |
| 5 | 2 | 3 |
| DISCERN | | |
| 1 | 7 | 13 |
| 2-3 | 60 | 43 |
| 4-5 | 27 | 39 |
| 6-7 | 5 | 4 |
| 8 | 1 | 1 |


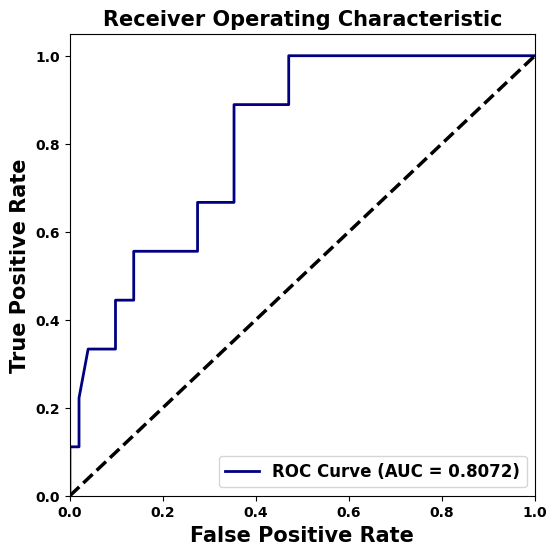


Figure S1. ROC-AUC Curve of the XGBoost Model.


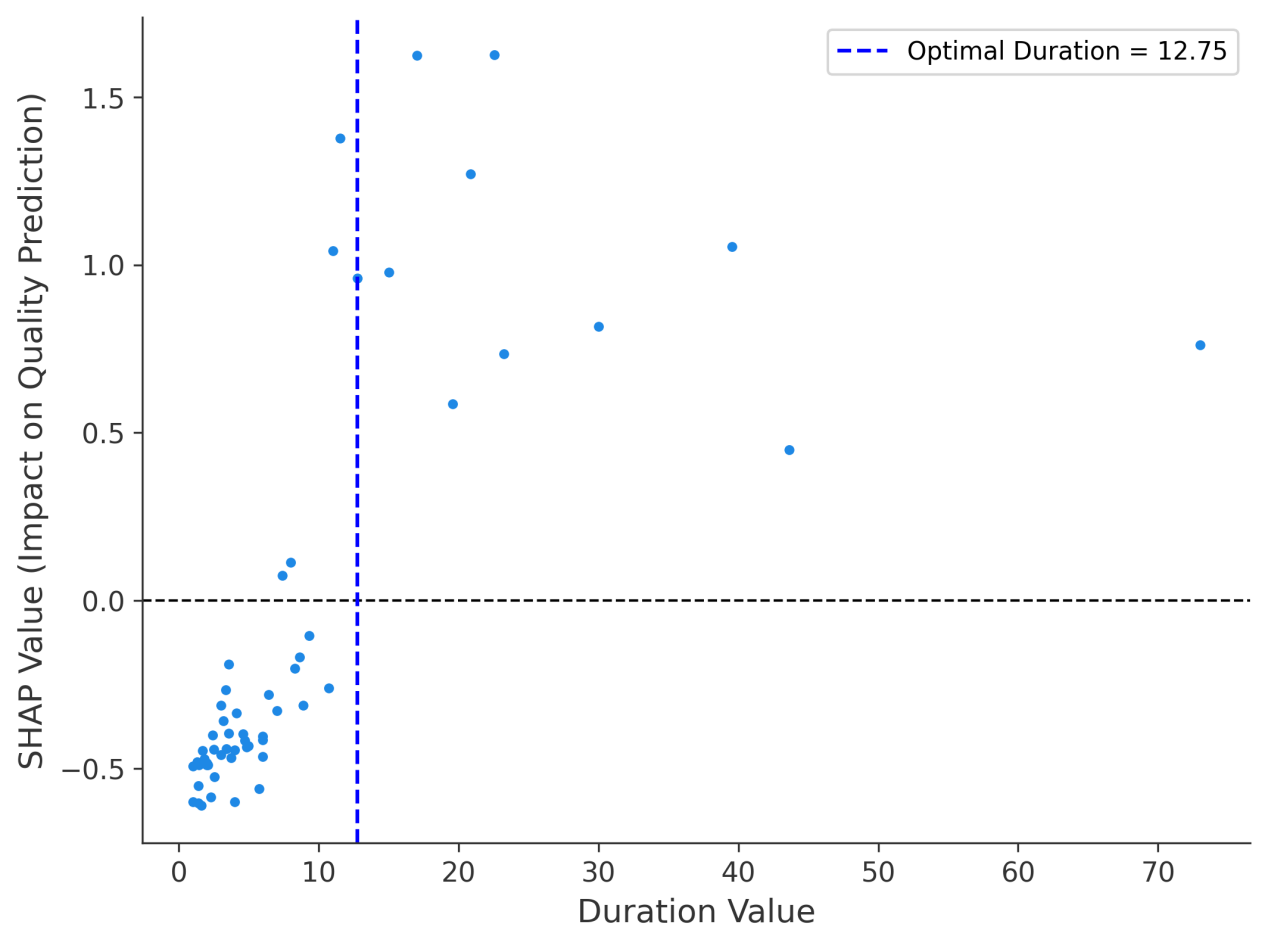


Figure S2. SHAP Dependence Plot Showing the Impact of Video Duration on Model Predictions.
